# Hyperemesis Gravidarum Presenting as Severe Hypokalemic Periodic Paralysis and Type II Respiratory Failure: A Different Form of Thyroid Storm?

**DOI:** 10.7759/cureus.19566

**Published:** 2021-11-14

**Authors:** Srinivas Naik, Dhruv Talwar, Sourya Acharya, Sunil Kumar, Deepti Shrivastava

**Affiliations:** 1 Department of Medicine, Jawaharlal Nehru Medical College, Datta Meghe Institute of Medical Sciences (Deemed to be University), Wardha, IND; 2 Department of Obstetrics and Gynaecology, Jawaharlal Nehru Medical College, Datta Meghe Institute of Medical Sciences (Deemed to be University), Wardha, IND

**Keywords:** hypercapnea, kir 2.6, beta hcg, hypokalemic periodic paralysis, thyrotoxicosis

## Abstract

Hyperthyroidism in pregnancy is a condition that results from an excess of beta-human chorionic gonadotropin hormone resulting in gestational thyrotoxicosis. This thyrotoxicosis of pregnancy might be linked with hyperemesis gravidarum and is usually a self-limiting disease. Hyperthyroidism can cause hypokalemic periodic paralysis, which presents as pure motor areflexic flaccid paralysis. In severe cases, it may involve respiratory muscles and cause hypercapnic respiratory failure requiring invasive ventilation. A positive feed-forward cycle of hypokalemia could be triggered by the loss of function of inward rectifier potassium channel 18 (Kir2.6) along with the increased activity of sodium, potassium-adenosine triphosphatase (Na⁺/K⁺-ATPase). Hyperthyroid periodic paralysis is characterized by biochemical hyperthyroidism, normal urine potassium excretion, and electrocardiography abnormalities.

We report a case of a 23-year-old female (G2P0L0A1) who had severe hyperemesis gravidarum and later on developed flaccid quadriplegia. Her thyroid profile revealed hyperthyroidism. She later developed hypercapnic respiratory failure and was managed by potassium replacement and invasive ventilation.

## Introduction

Hyperthyroid periodic paralysis (HPP) is a rare but potentially lethal manifestation of hyperthyroidism. It is characterized by acute paralytic attacks and hypokalemia in association with symptoms of hyperthyroidism [1]. The corpus luteum and the placenta generate the heterodimeric glycoprotein human chorionic gonadotropin (hCG). Early in pregnancy, hCG levels rise quickly and peak at about 10 weeks gestation, after which they fall until the third trimester, where they remain steady for the rest of the pregnancy. The beta subunit of hCG is structurally similar to thyroid-stimulating hormone (TSH), it can mildly stimulate the TSH receptor, enhancing thyroid hormone synthesis and contributing to the decreased TSH levels seen towards the end of the first trimester [2]. Hyperemesis gravidarum, a disorder linked to elevated hCG levels, is characterized by nausea and vomiting. In two-thirds of patients, overt biochemical hyperthyroidism (suppressed TSH, elevated free T4) is present, often in the absence of clinical symptoms of thyrotoxicosis, and resolves with the resolution of the hyperemesis [3,4]. The degree of hyperemesis is proportional to the degree of thyroid dysfunction.

Hypokalemia is one of the most prevalent disorders of fluid and electrolyte imbalance in clinical practice. Normal values of potassium in the blood lie between 3.5 and 5.5 meq/L. Definition of hypokalemia is a potassium level that is below 3.5 meq/L. Clinically, hypokalemia might present with weakness of the muscles and, in certain cases, arrhythmias. Inadequate intake of potassium linked with increased potassium loss through the route of skin, gastrointestinal tract, or kidneys and increased consumption of potassium as a result of increased cell production might result in hypokalemia. Low serum potassium may also be a result of alkalemia due to endogenous or iatrogenic exposure causing increased catecholamines or an increase in levels of insulin. This hypokalemia linked with alkalemia results from the intracellular shifting of potassium where the serum potassium decreases whereas total body potassium reserve remains intact. Hypokalemia occurs in around 1% of pregnancies [5].

In patients with thyrotoxicosis, HPP is defined by an initial onset of severe hypokalemia and substantial proximal muscle weakness [6]. HPP's pathophysiology is still unknown. Membrane excitability and muscular contractions are controlled by sodium, chloride, calcium, and potassium channels on cell membranes. Any of these cellular transport pathways, particularly the 3Na+/2K+-ATPase pump, may be disrupted, resulting in aberrant muscular contractibility and paralysis [7].

## Case presentation

A 23-year-old female patient (G2P0L0A1) came to the hospital with the complaint of vomiting for three months, weakness in all four limbs for four days, and altered sensorium for four days. The patient was apparently alright three months back and then she had sudden onset vomiting of around 10 to 15 episodes per day, containing food particles and nonprojectile in nature. There was no history of blood in the vomitus. The patient also had a history of weakness in all four limbs for four days along with altered sensorium. There was no history of fever, seizures, headache, giddiness, blurring of vision, diplopia, and diarrhea. There was no history of thyroid disorder in the past and the patient had no history of symptoms such as palpitations, weight loss, increased appetite, or heat intolerance suggestive of hyperthyroidism. There was no family history of thyrotoxicosis.

On examination, the general condition of the patient was poor, blood pressure was 130/80 mmHg, pulse was 155 beats per minute, and respiratory rate was 10 cycles per minute. Oxygen saturation while breathing ambient air was 92%. Signs of dehydration were present. There was no pallor, icterus, clubbing, cyanosis, edema, or lymphadenopathy. There was no goiter.

On CNS examination, the patient was stuporous with a Glasgow Coma Scale score of 7 (E2V1M4). The cranial nerves were intact. Head drop was seen on motor system examination, power was 1/5 in all four limbs, hypotonia was present in all four limbs, and generalized areflexia was present. Plantars were bilateral flexor. There were no signs of meningeal irritation. The cardiovascular and respiratory examination was normal. The patient was admitted to the medical intensive care unit with a probable diagnosis of periodic palsy.

**Figure 1:**
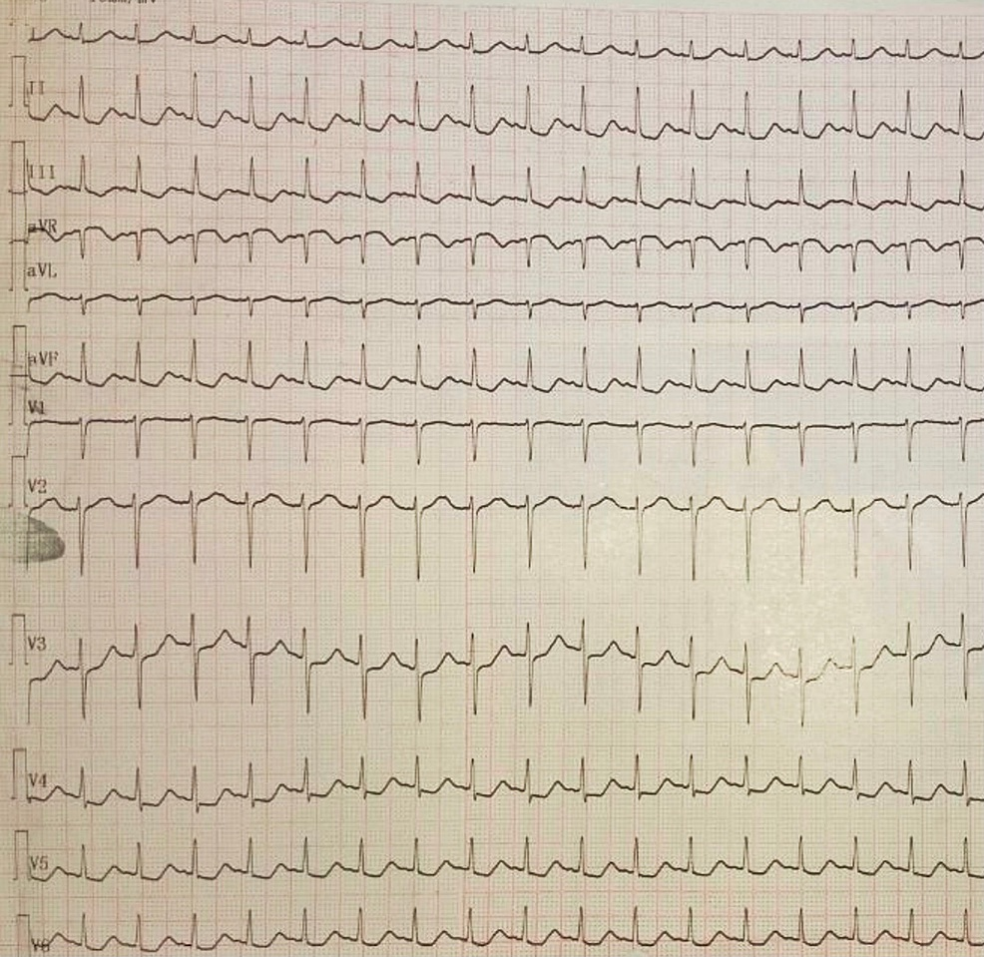
Electrocardiography with ST depression in lead II, III, V3, V4, V5, and V6.

Blood investigations of the patient are mentioned in Table 1. Arterial blood gas (ABG) revealed a pH of 7.12, partial pressure of carbon dioxide (pCO_2_) of 89 mmHg, partial pressure of oxygen (PaO_2_) of 80 mmHg, and bicarbonate (HCO_3_) level of 26 mmol/L, suggestive of respiratory acidosis. Urinary potassium was 19 meq/l. The urinary potassium to creatinine ratio was 1:5. The transtubular potassium gradient was calculated to be 7. Chest X-ray was normal. Electrocardiography revealed global ST-segment depression (Figure 1). A final diagnosis of hyperemesis gravidarum aggravated hyperthyroid periodic palsy was kept.

**Table 1:** Laboratory investigations of the patient.

Laboratory investigation	Measured value
Hemoglobin	10.4 g/dl (normal range 11.5 to 13.0 g/dl)
White blood cell count	13,500 cu/mm (normal range 4,500 to 11,000 cu/mm)
Platelet count	120,000/cumm (normal range 150,000 to 450,000)
Serum urea	39 mg/dL (normal range 5 to 20 mg/dl)
Creatinine	1.3 mg/dL (normal range 0.74 to 1.35 md/dl)
Sodium	149 mmol/L (normal range 135 to 145 mmol/l)
Potassium	1.8 mmol/L (normal range 3.5 to 5 mmol/l)
Serum glutamic-oxalacetic transaminase	32 units/L (normal range 8 and 45 units/l)
Serum glutamic-pyruvic transaminase	36 units/L (normal range 7 to 56 units/l)
Albumin	3.2 gm/dL (normal range 3.4 to 5.4 gm/dl)
Magnesium	2.2 mg/dl (normal range 1.7 to 2.2mg/dl)
Free T3	3.24 pg/ml (normal range 2.77 to 5.27 pg/ml)
Free T4	19 ng/dl (normal range 0.78 to 2.19 ng/dl)
Thyroid-stimulating hormone	0.015 milli-international units per liter (normal range 0.465 to 4.68 milli-international units per liter)
Anti-thyroid peroxidase antibody	Positive
Anti-thyroglobulin antibody	Negative
Anti-thyroid receptor antibody	Negative
International normalized ratio	1.27 (normal range 1.1 or below)
D-Dimer	699 ng/mL (normal range below 500 ng/ml)
Beta human chorionic gonadotropin	33,066 mIU/ml (normal range less than 35 mIU/ml)

The patient was treated with intravenous potassium replacement with injectable potassium chloride (160 meq given in first 24 hours and 160 meq given in the next three days), antithyroid drugs given were propylthiouracil 200 mg four times a day on day one tapered to 100 mg thrice a day from day two onwards and tablet propranolol 20 mg thrice daily through nasogastric (NG) tube, and intravenous thiamine 100 mg thrice a day. In view of decreased respiratory rate and severe type 2 respiratory failure (with pCO_2_ more than 50 mmHg and PaO_2_ less than 60 mmHg), intubation and mechanical ventilation were initiated. Obstetric consultation was taken. On the first day of admission, fetal heart and sounds were present. On day two of admission, the patient had intrauterine death. The fetus was evacuated by misoprostol infusion and cervical dilation.

Serum potassium was normalized by day four, the patient was maintaining saturation, ABG was normal, and the ventilator was weaned off. Power in limbs increased to grade 4/5. The patient was discharged on the eighth day with tablet methimazole 20 mg thrice a day and tablet propranolol 20 mg thrice a day. The patient is doing well on follow-up at present.

## Discussion

One of the most common causes of high-risk pregnancy is hyperemesis gravidarum. Hyperemesis gravidarum presents with persistent vomiting, which is not relieved despite the use of antiemetics [1]. It can be attributed to the hypersecretion of beta-hCG [4]. Prompt diagnosis of this condition is of utmost importance as it can not only be deleterious to fetal health but can also affect maternal health adversely. It can lead to not only electrolyte disturbances but can affect kidney function too.

Hypokalemia is a condition in which there is depletion of serum potassium. A level of serum potassium less than 3.5 mg/dl can be defined as hypokalemia [2]. Its manifestations include muscle weakness as well as cardiac complications such as QT prolongation, flattening of T wave, the appearance of U wave, and ST-segment depression. Causes for hypokalemia can be attributed to low potassium intake, loss of potassium from various sources such as persistent diarrhea, and loose stools leading to total depletion of body potassium and serum potassium. Persistent vomiting in hyperemesis gravidarum can cause severe hypokalemia [4]. Hypokalemia leading to muscle paralysis is a common occurrence. But hypokalemic paralysis occurs infrequently during pregnancy. Acute muscle weakness occurs in conjunction with low potassium levels. Any pregnancy that is linked to paralysis is considered high risk and should be treated as such. If a woman is experiencing major weakness or paralysis attacks, the related cardiac, respiratory, and muscular disorders may represent a risk, and she will need to be closely monitored and given informed treatment. A comprehensive cardiovascular examination is required. If an episode of weakness or paralysis occurs during labor and delivery, the medical team must be prepared to address it appropriately.

**Figure 2:**
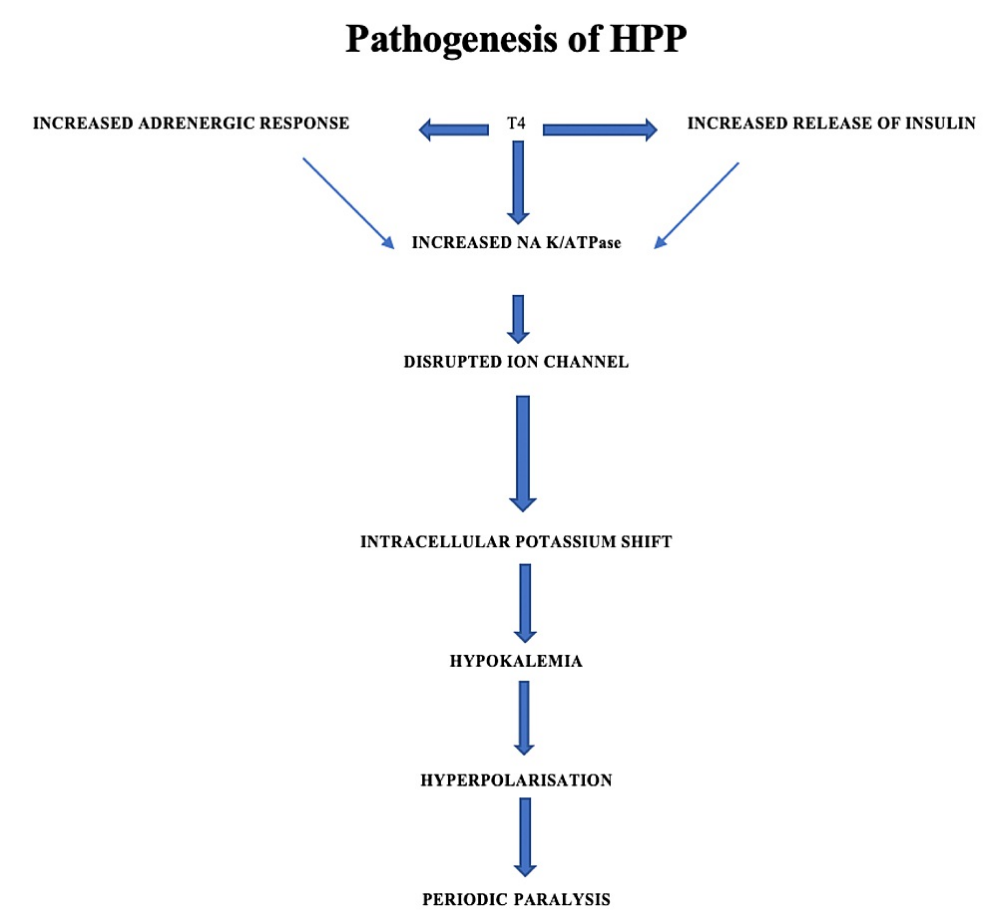
Pathophysiology of hyperthyroid periodic paralysis (HHP).

HPP is a rare but possibly fatal complication of hyperthyroidism that primarily affects young Asian males between the ages of 20 and 40 years, despite the fact that hyperthyroidism is more common in females. In addition to hyperthyroidism, it is characterized by acute paralytic episodes and hypokalemia. Quadriparesis is a rare complication that must be distinguished from Guillain-Barré syndrome, transverse myelitis, and spinal cord compression. Some patients may endure recurrent episodes of weakness in between attacks, although they will generally recover completely. The pathogenesis of HPP has been described in Figure 2 [7].

Renal causes of potassium loss were ruled out in our case through normal urinary potassium levels. The patient's blood pressure was also in the normal range; hence, causes like hyperaldosteronism and malignant hypertension were ruled out. Creatinine levels were monitored regularly and were within the normal range, ruling out acute kidney injury. Sodium and magnesium levels were also monitored throughout the hospital stay and were within the normal range.

In the above scenario, it is reasonable to state that thyrotoxicosis induced hypokalemia in our case.

Our case was essentially important as hyperthyroidism, which was probably precipitated by pregnancy, had serious manifestation in the form of HPP. Although hyperthyroid has been reported previously to cause paralysis, this case was exceptional as it was linked with pregnancy and hyperemesis gravidarum, which is rare [8]. This paralysis required mechanical ventilation and ultimately caused the intrauterine demise. However, maternal mortality was prevented and the patient was treated successfully with a team approach of physicians and obstetricians. Thus, it is essential to diagnose and treat HPP promptly, especially in high-risk states, such as pregnancy, to prevent mortality.

## Conclusions

Hyperthyroid periodic paralysis in a case of hyperemesis gravidarum is a rare entity and needs to be treated with utmost vigilance as it can not only help in saving the life of the mother but also of the fetus as well. It can be a life-threatening condition; hence, early diagnosis and prompt treatment are of dire need. More research needs to be directed into this rare entity as it will educate the treating physicians and obstetricians and thus help in reducing maternal mortality.
